# Extensive Brainstem Posterior Reversible Encephalopathy Syndrome in a Hemodialysis Non-Adherent Patient

**DOI:** 10.7759/cureus.14523

**Published:** 2021-04-16

**Authors:** Ya Haddy Sallah, Adeel S Zubair, Jeffrey J Dewey

**Affiliations:** 1 Neurology, Yale School of Medicine, New Haven, USA

**Keywords:** radiological findings in pres, brainstem lesion, hypertension, seizures, pres, posterior reversible encephalopathy syndrome (pres), systemic hypertension

## Abstract

Posterior reversible encephalopathy syndrome (PRES) refers to a disorder of reversible vasogenic edema caused by rapid hyperperfusion of the brain that classically involves areas supplied by the posterior circulation such as the parieto-occipital region. It may present with atypical features such as brainstem and spinal cord involvement. Common causes include renal failure, pre-eclampsia/eclampsia among pregnant women, rapid changes in systemic blood pressure, and autoimmune diseases. The most prevalent presenting signs and symptoms are encephalopathy, seizures and headache. A 64-year-old female presented to a dialysis unit after missing several sessions with twitching in her extremities and elevated blood pressure. Additionally, she recently terminated clonidine use and was likely experiencing rebound hypertension. The continuous electroencephalogram (EEG) demonstrated generalized, non-convulsive seizures. MRI findings were notable for hyperintensities in the pons, middle cerebellar peduncles, cerebellar hemispheres, and periventricular and subcortical matter with medulla and proximal spinal cord involvement. A notable clinical sequela of PRES in this patient was coma. Aggressive blood pressure control led to significant improvement and return to her neurologic baseline. PRES can present with extensive brainstem involvement with a clinical sequela of coma. Multiple underlying causes such as dialysis non-adherence and rebound hypertension following clonidine discontinuation contributed to the development of this condition in this patient.

## Introduction

Posterior reversible encephalopathy syndrome (PRES) is a disorder of reversible subcortical vasogenic edema classically involving the parieto-occipital region on brain imaging. Known causes of PRES include renal failure (55% of cases), sudden changes in blood pressure, pre-eclampsia/eclampsia, cytotoxic drugs, and autoimmune diseases [1]. The leading theory on the pathophysiology of PRES is the rapid development of hypertension which exceeds the capacity of cerebral blood flow autoregulation resulting in hyperperfusion and subsequent breakdown of the blood-brain barrier through cytokine release. This leads to increased interstitial extravasation and brain edema [1,2]. Brain areas supplied by the posterior circulation are considered particularly susceptible to hyperperfusion due to limited sympathetic innervation [1]. Other regions such as the frontal and temporal lobes, cerebellum, basal ganglia and brainstem can be involved, however, this is frequently accompanied with changes in the parieto-occipital region [1-4]. The most common presenting signs and symptoms of PRES include encephalopathy, seizures, headaches, visual disturbances, focal neurologic deficits, and status epilepticus [1].

## Case presentation

A 64-year-old female with a past medical history of end-stage renal disease on hemodialysis, hypertension, and ischemic stroke with left-sided residual weakness presented to dialysis after missing three sessions. While there, she was noted to be lethargic and had twitching in her extremities. Vital signs were notable for blood pressure of 207/110 mmHg. Laboratory findings were notable for mild macrocytic anemia, low estimated glomerular filtration rate (22 ml/min), and negative urine drug screen. She had what was described by an outside hospital as a generalized convulsive seizure following which she was intubated and received a loading dose of levetiracetam. Continuous electroencephalogram (EEG) was on the ictal-interictal continuum, demonstrating extended runs of generalized periodic discharges with over-riding rhythmicity as well as generalized rhythmic delta activity with brief periods meeting criteria for non-convulsive seizures, which responded to phenytoin after no improvement with levetiracetam. Despite electrographic improvement, she was arousable only to significant noxious stimuli and did not follow commands. Her gaze was conjugate and pupils sluggishly reactive to light. Her bilateral corneal, oculocephalic and cough reflexes were intact. A motor examination was notable for triple flexion in her right lower extremity, subtle withdrawal in the left lower and bilateral upper extremities. Reflexes were 2+ in the upper extremities and absent in the lower extremities. Babinski sign was positive bilaterally. She also had a bilateral Hoffman’s sign. 

**Figure 1:**
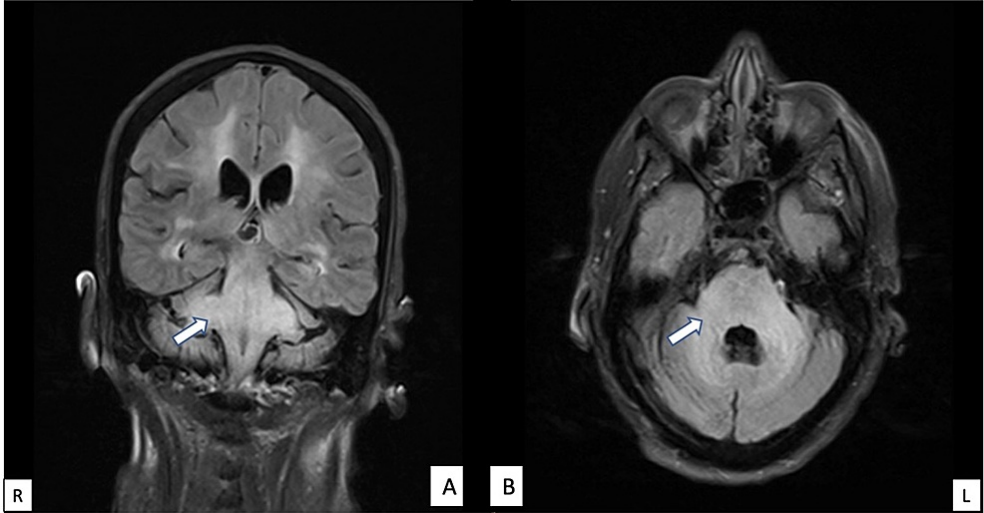
Extensive FLAIR hyperintensities are demonstrated within the subcortical and periventricular white matter, brainstem, middle cerebellar peduncles and cerebellar hemispheres, with moderate improvement of T2/FLAIR hyperintense signal within the brainstem: (A) coronal view, (B) axial view. FLAIR: fluid-attenuated inverted recovery.

Initial head CT scan demonstrated hypodensities concerning for brainstem and cerebellar white matter edema in a diffuse and symmetric pattern. A brain MRI demonstrated T2-weighted fluid-attenuated inverted recovery (FLAIR) confluent hyperintensities in the pons, bilateral middle cerebellar peduncles (with associated restricted diffusion), cerebellar hemispheres, periventricular and subcortical matter, extending down to the medulla and proximal spinal cord (Figure 1). Patchy hyperintense signals were also seen in the bilateral corona radiata and left insula with restricted diffusion. Magnetic resonance angiography (MRA) was negative for any abnormalities. A lumbar puncture was not performed due to refusal by the power of attorney.

Based on the MRI findings, the differential diagnosis included PRES secondary to hypertension, infarcts secondary to basilar occlusion, metabolic encephalopathy, and infectious encephalitis. Given the concern for PRES and no evidence of large arterial occlusion, she was initiated on aggressive blood pressure control with a systolic blood pressure goal of less than 180 mmHg with hydralazine, carvedilol, and lisinopril. Blood pressure goal was achieved with isolated systolic blood pressure spikes above 200 mmHg. A repeat MRI four days later showed mild improvement in the T2-weighted/FLAIR hyperintensities. By this time, her mental status had started to demonstrate signs of improvement, suggesting gradually reversing pathology. By day eleven of admission, she was at her neurological baseline with continued chronic left-sided deficits.

## Discussion

This case demonstrates multiple atypical features of PRES. First, though brainstem and cerebellar involvement are present in a minority of cases, additional rostral spinal cord involvement is rare [5]. Second, while the majority of patients with PRES have vasogenic edema, cytotoxic edema was also present in this case, evidenced by restricted diffusion in the middle cerebellar peduncles, corona radiata, and left insula. Cytotoxic edema may be reversible, but can progress to infarction on follow up [2]. Diffusion restriction also suggests a poor prognosis [2]. Using the criteria established by Schweitzer et al. 2017, the imaging findings meet criteria for (1) extensive vasogenic edema, and (2) advanced radiologic PRES (at least one of the following: extensive edema, diffusion restriction or hemorrhage with mass effect), which were associated with poorer clinical outcomes [6].

Our patient developed atypical PRES in the setting of hypertension secondary to multiple etiologies: noncompliance with hemodialysis and rebound hypertension after discontinuation of clonidine patches. She had missed three dialysis sessions which likely contributed to her presentation. Renal failure is one of the most common causes of PRES and isolated brainstem and cerebellar PRES has been reported in patients non-adherent to hemodialysis [1,5,7]. A recently published study also found that majority of patients presenting to the emergency department with PRES were hypertensive similar to the patient described in this case [8]. 

Common presenting symptoms of PRES include encephalopathy, seizures and headache [1]. This case illustrates a seizure followed by non-convulsive status epilepticus as a possible presenting symptom, which is uncommon [1]. It is possible that PRES may have been a consequence rather than the cause of a seizure, however, there was no localization on EEG and no other identifiable etiology for seizures were indicated in the history or laboratory workup. Significant brainstem involvement in this patient also led to the clinical sequelae of coma, presumably due to extensive disruption of the reticular activating system. While infectious etiologies could not be definitively ruled out because of refusal of the power of attorney for a lumbar puncture, there were no systemic signs of infection and her rapid spontaneous improvement would not be consistent with active infection.

## Conclusions

This case report adds to the growing literature on brainstem variants of PRES, notably with spinal cord involvement and advanced radiologic PRES that clinically resolved with aggressive blood pressure control. PRES should be on the differential in patients with renal failure who present with changes in mental status, particularly if they are overdue for hemodialysis. Prompt neuroimaging can identify PRES and expedite management. Atypical imaging findings warrant thorough workup for other diagnoses, though as this case illustrates, findings isolated to the posterior fossa and rarely involving the rostral cervical spinal cord can be seen.
